# Applicability of a pathological complete response magnetic resonance-based radiomics model for locally advanced rectal cancer in intercontinental cohort

**DOI:** 10.1186/s13014-022-02048-9

**Published:** 2022-04-15

**Authors:** Luca Boldrini, Jacopo Lenkowicz, Lucia Clara Orlandini, Gang Yin, Davide Cusumano, Giuditta Chiloiro, Nicola Dinapoli, Qian Peng, Calogero Casà, Maria Antonietta Gambacorta, Vincenzo Valentini, Jinyi Lang

**Affiliations:** 1 Diagnostica per Immagini, Radioterapia Oncologica ed Ematologia, Fondazione Policlinico Universitario “A. Gemelli” IRCCS, Rome, Italy; 2 Department of Radiation Oncology, Sichuan Cancer Hospital and Institute, School of Medicine, University of Electronic Science and Technology of China, Chengdu, China

**Keywords:** Radiotherapy, Radiomics, Rectum, Magnetic resonance imaging, Intensity modulated radiation therapy

## Abstract

**Background:**

Predicting pathological complete response (pCR) in patients affected by locally advanced rectal cancer (LARC) who undergo neoadjuvant chemoradiotherapy (nCRT) is a challenging field of investigation, but many of the published models are burdened by a lack of reliable external validation. Aim of this study was to evaluate the applicability of a magnetic resonance imaging (MRI) radiomic-based pCR model developed and validated in Europe, to a different cohort of patients from an intercontinental cancer center.

**Methods:**

The original model was based on two clinical and two radiomics features extracted from T2-weighted 1.5 T MRI of 161 LARC patients acquired before nCRT, considered as training set. Such model is here validated using the T2-w 1.5 and 3 T staging MRI of 59 LARC patients with different clinical characteristics consecutively treated in mainland Chinese cancer center from March 2017 to January 2018. Model performance were evaluated in terms of area under the receiver operator characteristics curve (AUC) and relative parameters, such as accuracy, specificity, negative and positive predictive value (NPV and PPV).

**Results:**

An AUC of 0.83 (CI 95%, 0.71–0.96) was achieved for the intercontinental cohort versus a value of 0.75 (CI 95%, 0.61–0.88) at the external validation step reported in the original experience. Considering the best cut-off threshold identified in the first experience (0.26), the following predictive performance were obtained: 0.65 as accuracy, 0.64 as specificity, 0.70 as sensitivity, 0.91 as NPV and 0.28 as PPV.

**Conclusions:**

Despite the introduction of significant different factors, the proposed model appeared to be replicable on a real-world data extra-European patients’ cohort, achieving a TRIPOD 4 level.

**Supplementary Information:**

The online version contains supplementary material available at 10.1186/s13014-022-02048-9.

## Background

Medical imaging plays to date a crucial role in tumour staging, clinical workflow definition and prognostic stratification of oncological patients. Radiological findings, integrated with histopathology, blood exams and numerous other clinical parameters, usually determine the optimal therapeutic strategy for each patient affected by a tumour [1]. The opportunity to take fully advantage of quantitative parameters extracted from medical imaging, could provide a more comprehensive characterisation of the tumour, suggesting a more tailored clinical pathway [2]. In the field of quantitative analysis of medical imaging, radiomics represents a topic of growing interest, relying on the biological assumption that the tumours properties (biological aggressiveness) could be described by the phenotypic heterogeneity, and radiological images could allow to successfully quantify it, paving the way to more effective approaches in terms of treatment personalization [3].

Unfortunately, radiomics features are still burdened by several methodological and biological vulnerabilities that hamper their fully aware and effective integration in multidimensional clinical interpretation and translational applications [4, 5].

Rectal cancer represents a significant field of application for radiomics [6], as several grey areas still persist in the management of these patients [7], especially considering the complex interaction among different specialties required to define the optimal therapeutic approach [8, 9]. Neoadjuvant radio-chemotherapy (nCRT) followed by total mesorectal excision (TME) is the current standard of care for locally advanced rectal cancer (LARC) [10, 11] patients and presents variable tumour responses, with nearly 20–30% of pathological complete response (pCR).

Different experiences highlighted that patients showing pCR generally show favourable survival outcomes [12, 13]. The definition of new parameters able to early predict the biological behaviour of rectal cancer in terms of response to the neoadjuvant treatments, represents an innovative strategy to better define organ preservation and less invasive surgical approaches, especially for patients who have overall good prognostic factors [14–16].

Even if promising evidence has been produced so far, radiomics still does not represent the standard to characterise rectal cancer behaviour and a growing number of studies is oriented to identify and define a radiomic signature able to predict clinical outcomes [17–20] or response to neoadjuvant treatment [20–23]. Promising advances about the role of radiomic biomarkers have also been made thanks to the recent introduction of hybrid MR radiotherapy delivery units (MR-Linac) that make available unprecedented quantities of images [24–26].

A robust methodology needs to be pursued in order to allow a full integration of radiomic tools into clinical practice, and existing models need to be validated using external cohorts of patients with the aim to test their replicability [27].

To ensure the reliable development and validation of predictive models, the international scientific community recently established a group of recommendations, known as TRIPOD statements, proposing a standardized methodology where the independent model validation using an external dataset plays a crucial role. Within this framework, also radiomics studies need to fully meet the TRIPOD requirements [22, 28]. The aim of this study was to evaluate the replicability of a radiomic model for pCR prediction, developed and firstly validated in Europe (as TRIPOD 3), on a cohort of patients enrolled in an Asian cancer centre having different clinical characteristics and following different treatment workflows, therefore reaching a TRIPOD 4 evidence score [20].

## Methods

### Radiomic model for pCR prediction

The magnetic resonance imaging (MRI) based radiomics model object of this study aimed to early identify patients who will undergo pCR. This vendor-independent model was set up in Italy using a single-center training set composed by 162 patients and an external validation set composed by 59 cases provided by two other European centers. All the patients included in the training and validation cohorts underwent diagnostic 1.5 T MRI prior to the neoadjuvant chemoradiotherapy (nCRT) treatment.

External beam radiation therapy (EBRT) was delivered to the whole mesorectum and the drainage nodal stations (total dose of 45 Gy, 1.8 Gy per fraction) and a boost of 50.4 or 55.0 Gy to the gross tumour volume (GTV), if delivered with sequential boost or simultaneous integrated boost respectively. Patients then underwent TME surgery 8–12 weeks after the end of nCRT. Different concomitant chemotherapy regimens were allowed: oral capecitabine 1650 mg/m^2 ^* die (d1-7, q7); 5-fluorouracil 225 mg/m^2 ^* die (d1-7, q7) or CapOx 60 mg/m^2^ of iv oxaliplatin (d1, q7) plus oral capecitabine 1300 mg/m^2 ^* die (d1-7, q7) according to staging and general conditions of the patients.

Response to nCRT was evaluated by histopathological examination of surgical resected specimens: tumour responses were classified using tumour regression grade (TRG) according to Mandard et al. [29] and pCR was defined as the absence of tumour disease on surgical specimen (ypT0N0).

The radiomics analysis was performed using standard staging MR images acquired before the start of nCRT.

Radiomic features were extracted from T2-weighted MR images as suggested in Dinapoli et al. after the application of the Laplacian of Gaussian (LoG) filter. In particular the entropy was calculated using a kernel width (σ) equal to 0.344 mm, while the skewness using a σ of 0.485 mm [20].

The significant features and the corresponding coefficient values of the linear regression logistic model are summarised in Table 1.

**Table 1:** Parameters adopted in the original model (Dinapoli et al., 2018), object of validation in this study

Parameter	Filter (mm)	Coefficient	SD
Intercept	NA	− 6.18	3.00
cT	NA	− 0.95	0.36
cN	NA	0.53	0.35
Skewness^a^	LoG (0.48)	− 3.01	1.17
Entropy^a^	LoG (0.34)	3.61	1.68

^a^LoG filter’s kernel width is reported in millimetres for the two radiomics features

The multivariable model obtained in [20] was based on four covariates: two clinical parameters (clinical tumour and nodal staging, cT and cN) and two radiomics features (skewness and entropy). Its stability was confirmed on both internal and external validation cohorts showing an area under curve (AUC) receiver operating characteristic (ROC) values of 0.73 (95% CI, 0.65–0.82) and 0.75 (CI 95%, 0.61–0.88), respectively. For further details regarding the MRI radiomics-based model set up and results obtained in the training and validation steps, we address the reader to the previous publication [20].

### Intercontinental cohort workflow

Patients affected by pathologically proven LARC and treated at Sichuan Cancer Hospital between March 2017 and January 2018 were retrospectively enrolled in this validation study.

Inclusion criteria were: pathologically proven LARC, absence of artefacts in the pelvic MR staging image and availability of tumour regression grade (TRG) classification in the pathologic report.

Patients with distant metastases at diagnosis, younger than 18 years or denying informed consent for retrospective data collection, were excluded from the study.

All the patients meeting the inclusion criteria and consecutively treated during the period under investigation were considered for this study.

All the patients underwent diagnostic MRI with two different scanners available in the radiation oncology department: a Siemens Magnetom Skyra and a Siemens Magnetom Avanto systems (Siemens Medical Systems, Erlangen, Germany), having a static magnetic field strength B_0_ of 3 Tesla (27 patients), and 1.5 Tesla (32 patients), respectively.

T2-weighted fast spin echo 2D-oblique images acquired on a transversal plan orthogonal to the tumour longitudinal axis were used for the radiomics analysis, in order to be consistent with the original experience reporting the model.

The GTV was firstly contoured by a radiation oncologist of the lower gastro intestinal malignancies department, and then an independent validation was performed by a senior radiation oncologist of the same department.

After an initial cycle of chemotherapy CapOx lasting about 3 weeks and foreseeing capecitabine 1000 mg/m^2^ at d1-14 concurrently with oxaliplatinum 130 mg/m^2^ d1, patients followed two different treatment schedules prior to TME. The first treatment scheme involved a 1-week short course of external beam radiotherapy (EBRT, 25 Gy in 5 fractions of 5 Gy per fraction) followed directly after 1-week gap by TME; the second treatment scheme involved nCRT administering EBRT for 5–6 weeks (50.4 Gy in 28 fractions of 1.8 Gy each) concurrently with chemotherapy (Capecitabine 825 mg/m^2^ die), at the end of which two more cycle of CapOx were foreseen.

### Evaluation of the model performance

The clinical and treatment parameters of the intercontinental patients-cohort were compared with those reported in the previous experience [20] using the Mann Whitney test for continue variables and the chi-square test for categorical ones [23, 28–30].

The in house developed radiomics platform MODDICOM [25, 31] used to build the model, was used to process the MR images of the Asian cohort, applying the LoG filter and extracting the radiomic features required by the radiomic model.

Response to nCRT was determined following the same procedure adopted in the training cohort of LARC patients [20], both in terms of tumour classification and definition of complete responders.

The evaluation of the model performance was performed in terms of the area under the curve (AUC) of the receiver operator characteristics (ROC) curve; accuracy sensitivity, specificity, negative and positive predictive value (NPV and PPV) calculated at the optimal threshold identified considering the Youden Index on the original data reported. The statistical analysis was carried out using R software (version 3.3.1).

## Results

59 patients were lastly enrolled.

The patient characteristics of both the original cohort (European patients, used for training and validation of the model) and of the new cohort enrolled in the Sichuan Cancer Hospital are reported in Table 2.

**Table 2:** Clinical and treatment characteristics of the patient’s cohort

	Training^a^ [6]	Validation^b^ (present study)	Difference significance (*p* value)
Number	162	59	
Age—^c^ yr			
Median	65.0	56.0	0.67
Range	28.0–83.0	34.0–75.0	
Sex—no. (%)			
Male	123 (75.9)	47 (79.7)	0.56
Female	39 (24.1)	12 (20.3)	
T stage—^d^ no. (%)			
T2	15 (9.3)	6 (10.2)	0.98
T3	95 (58.6)	34 (57.6)	
T4	52 (32.1)	19 (32.2)	
N stage—^d^ no. (%)			
N0	9 (5.6)	25 (42.4)	< 0.05
N1	58 (35.8)	24 (40.7)	
N2	95 (58.6)	10 (16.9)	
MR scanner strength^e^			
1.5 T no (%)	162 (100.0)	32 (54.2)	< 0.05
3.0 T no (%)	–	27 (45.8)	
Interval MRI^e^ and start CRT^f^-mo^e^			
Median (range)	1.4 (0.0–10.0)	0.9 (0.7–1.0)	< 0.05
Interval end CRT^f^ and surgery-mo^g^			
RT short course: median/range	–	0.3 (0.3–0.5)	< 0.05
RT long course: median/range	2.6 (1.1)	1.9 (1.0–2.7)	
RT course—no (%)^h^			
Short (5fr × 5 Gy)	–	19 (32.2)	< 0.05

Statistical tests results investigating significant differences are reported in the last column: chi-square test was performed for categorical variables, Wilcoxon Mann Whitney for continuous ones

^a^Cohort 1: European Cohort, used for the training and first validation of the model

^b^Cohort 2: Intercontinental cohort

^c^*yr* years

^d^*no.* number

^e^*MR/MRI* magnetic resonance/magnetic resonance imaging

^f^*CRT* chemoradiotherapy

^g^*mo.* months

^h^*RT* radiotherapy

The original and new cohort of patients reported different magnetic field strength of the MR scanner used for imaging, different nCRT treatment schedules, with subsequent different interval between MRI and CRT and the end of CRT and surgery; no statistical differences in the patients age (*p* = 0.67), sex (*p* = 0.56), T staging (*p* = 0.98) were observed.

The proportion of T staging and sex are very similar between training and validation set, while the median age of validation cases (56 years, range 34–75) is lower than the corresponding training ones (65, range 28–83). No difference has been recorded for N stage.

The total number of pCR cases observed in the Chinese cohort was 10, corresponding to a pCR rate of 16.9% (10/59).

**Fig. 1:**
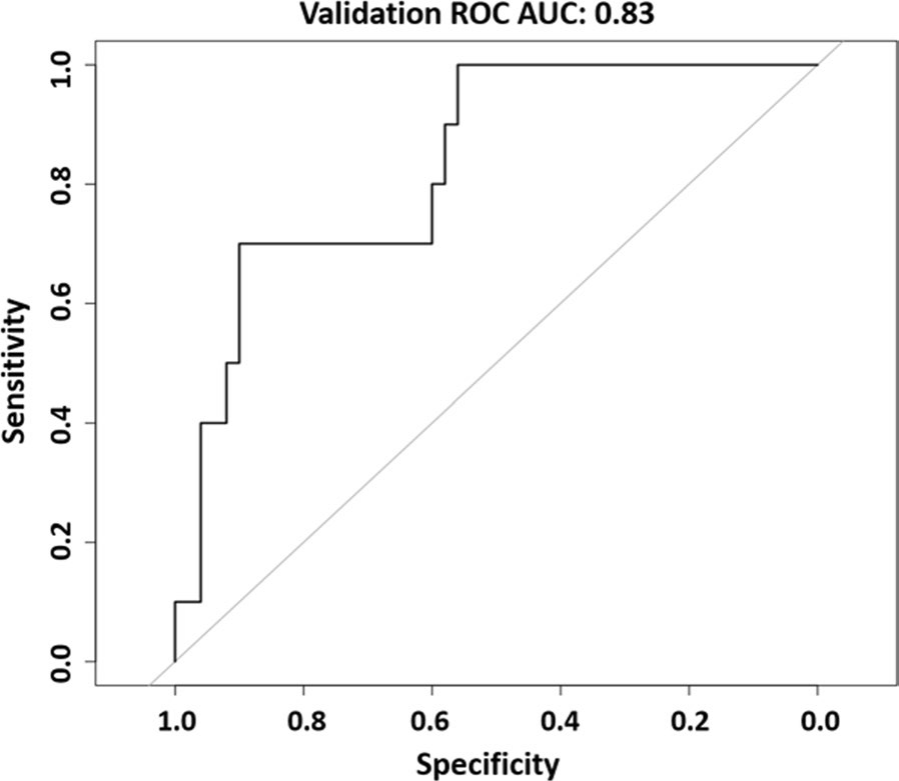
ROC curve obtained for the entire cohort of intercontinental patients

**Fig. 2:**
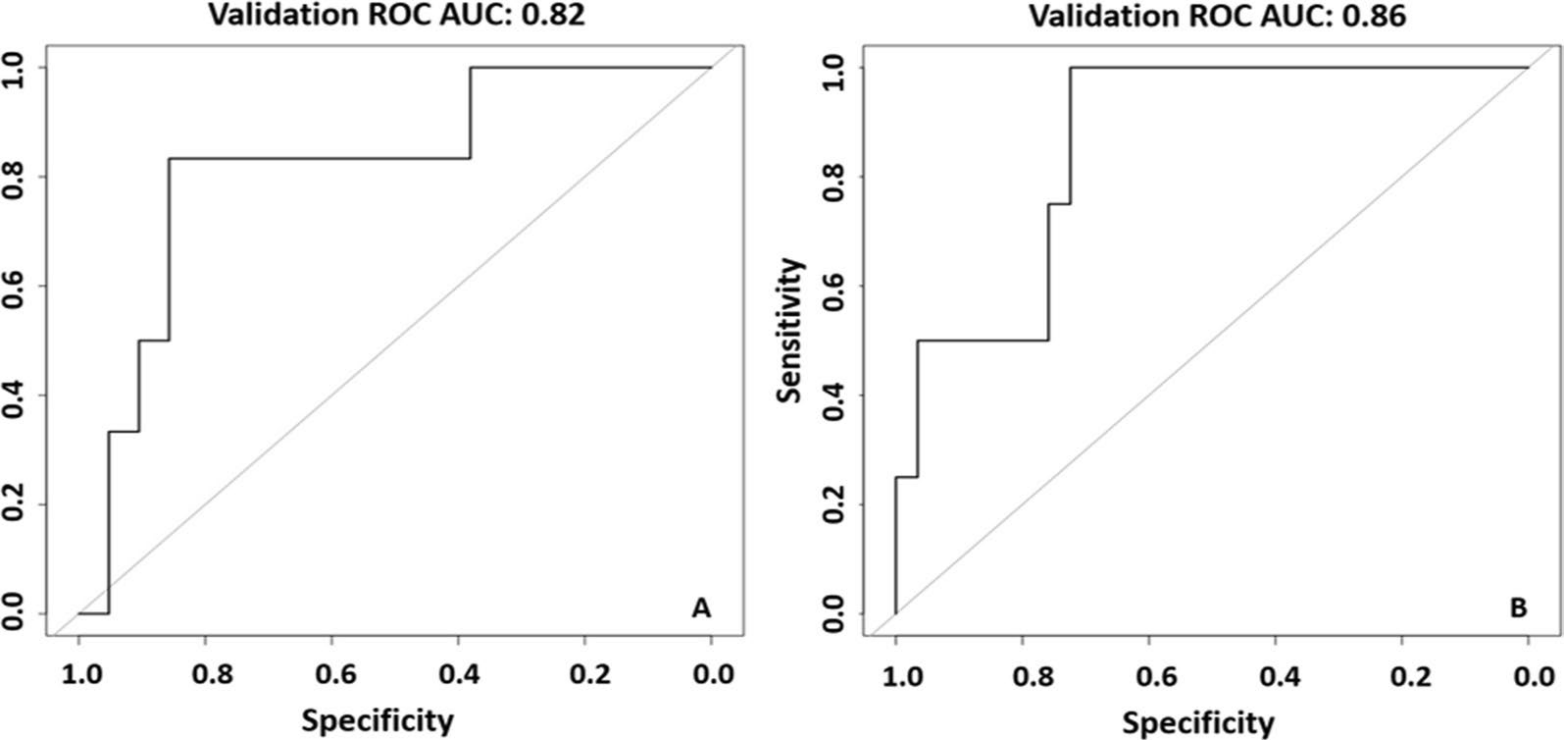
ROC curves and corresponding 95% CI, obtained for the intercontinental cohort of patients. Patients are imaged with 1.5 T (**A**) and 3.0 T (**B**) MRI scanners. The area under the curve (AUC) value is reported for each curve

When analysing the intercontinental cohort, the model reported a ROC curve with an AUC value of 0.83 and a 95% confidence interval of 0.71–0.96. The ROC curve is shown in Fig. 1. The applicability of the model tested analysing separately the patients of the intercontinental cohort imaged with a 1.5 T and 3.0 T scanner, gave AUC 0.82 (95% CI 0.61–1.00) and 0.86 (95% CI 0.70–1.00), respectively; the ROC curves obtained are reported in Fig. 2.

Moreover, the best cut-off value was 0.267 in the original experience, corresponding to a Youden index of 0.39 (specificity: 0.63, sensitivity: 0.76); the predictive performance observed for the intercontinental patient dataset considering this threshold are reported in Table 3.

**Table 3:** Predictive performance of the model applied to the intercontinental patients’ cohort with corresponding 95% confidence intervals

Parameter	Coh-whole^	Coh-1.5 T^^	Coh-3.0 T^^
Accuracy	0.65 (0.52–0.77)	0.52 (0.32–0.71)	0.76 (0.55–0.87)
Specificity	0.64 (0.49–0.77)	0.42 (0.22–0.66)	0.75 (0.56–0.90)
Sensitivity	0.70 (0.35–0.93)	0.83 (0.36–0.99)	0.50 (0.07–0.93)
NPV*	0.91 (0.80–0.97)	0.90 (0.59–0.98)	0.92 (0.80–0.97)
PPV*	0.28 (0.18–0.40)	0.29 (0.20–0.41)	0.22 (0.08–0.48)
Kappa statistics	0.22 (0.00–0.43)	0.15 (− 0.08 to 0.40)	0.17 (− 0.18 to 0.51)

^ whole cohort; ^^ patients that underwent 1.5 Tesla and 3.0 Tesla magnetic resonance imaging. *Negative and positive predictive values (NPV and PPV, respectively)

Relative confusion matrices are available as Additional file 1.

## Discussion

The predictive role of MRI in rectal cancer has been widely investigated [32] but, to the best of our knowledge, the introduction of radiomic models in clinical practice is still limited, mainly due to the lack of the independent validation studies.

Omics studies in general, and radiomic ones particularly, specifically lack ethnographic information in the models and the possibilities to test this aspect are pretty uncommon, leaving this interesting question unresolved. As far as the authors know, this study represents the first experience of external validation of a rectal cancer radiomic model on an extra-European population, while a previous validation on Asian cohort of patients has been performed for predictive models not including radiomics, with good results in terms of generalisation and overall stability [33].

During the last years, several authors tested MRI radiomics to evaluate its reliability in terms of nCRT response prediction for rectal cancer, including also the analysis of MR images acquired during or at the end of the course of treatment, investigating how the quantitative analysis can support clinicians in the choice of the optimal therapeutic strategy [34–41]. It should be noted that these models were generally trained and validated on very homogeneous clinical trial populations, reaching in the best-case scenario a TRIPOD 3 robustness score, with their possible generalization to a world population being still far to be thoroughly described [42–44].

This innovative external intercontinental validation experience has been realised on real-world imaging data of patients enrolled in a different continent, with MRI scans acquired on scanners characterised by different vendor and field strength with respect to those reported in the original model, significantly increasing data heterogeneity and critically stressing the original two-radiomics features model.

High predictive values were reported on the whole external dataset (AUC = 0.83, NPV 0.91), representing a significant result in the context of radiomics-models replicability and translational robustness. Interestingly, the relative subgroup analysis (AUC 0.82 on 1.5 T and 0.86 on 3 T patients) suggests that magnetic field intensity variability can be overcome by means of selecting appropriate image features, as also reported in a previous study [23].

It has to be noted that the PPV observed in the whole cohort is low 0.28 (0.18–0.40), with slightly better results for the 1.5 T cases (0.29, range 0.20–0.41) if compared to the 3 T ones, which reached only 0.22 (0.08–0.48).

This results could be explained by the limited absolute number of pCR in the Chinese cohort and the original design of the Dinapoli et al. model, set on 1.5 T images. Nevertheless, the very high NPV 0.91 (0.80–0.97) observed in the whole cohort represents a stimulating result in order to guide a more tailored treatment approach in patients predicted as “not responding” to nCRT. Similar experiences represent the rationale for new radiomics driven trials, where dose intensification is foreseen for patients predicted as not responding [41, 45].

Futhermore, the observed results suggest the applicability of the original model by Dinapoli et al. also for LARC patients undergoing short course radiotherapy, supporting the hypothesis of an intrinsic biological signature of the tumor that could be correlated with different radiomic features [20].

Besides the limited patients sample for the intercontinental validation (59 patients, representing the 36.4% of the original model sample), one of the possible limitations of this study maybe identified in the lack of a multi observer based segmentation to test the robustness of radiomic features, to which we preferred an expertise based segmentation (revision by a senior staff member). Nevertheless, this choice reflects the fact that decisions are generally taken on single operator’s segmentations in daily clinical practice and that the model object of this study is based on two first order radiomic features, which were recently categorised as the least sensitive to manual tumour delineation in a recent experience performed on MRI in LARC patients [46].

Despite the heterogeneity of the treatments offered to patients of the different cohorts (i.e. type of neoadjuvant treatment; EBRT administered in short versus long course; different MR vendors and field strength used; patient clinical and ethnic characteristics), the proposed model appeared to be overall replicable on a real-world data extra-European patients’ cohort, offering an innovative hint for radiomics validation procedures.

New experiences including larger cohorts of patients are encouraged to further validate the proposed model with tighter confidence intervals, confirming the generalizability of the model to the entire world population.

The next validation step will be represented by the setup of a prospective study in order to confirm the advantages of using these innovative imaging based predictive models supporting clinical decision making and personalised cancer care especially considering the innovative applications of MRI in radiation oncology, thanks to the promising applications of MR-Linacs in the management of rectal cancer [47].

## Conclusion

The intercontinental external validation of this model confirmed the robustness and replicability of the original model with a TRIPOD 4 level. The promising results obtained encourage further investigations for the application of radiomics modelling in the framework of complex multivariable decision support systems for LARC.

## Supplementary Information

**Additional file 1**. Confusion matrices of the predictive performances for the intercontinenental cohort.

## Data Availability

The data are fully available and will be included within the article and in its additional files.

## Abbreviations

pCR: Pathological complete response
LARC: Locally advanced rectal cancer
nCRT: Neoadjuvant chemoradiotherapy
MR: Magnetic resonance
AUC: Area under the curve
ROC: Receiver operating characteristics
NPV: Negative predictive value
PPV: Positive predictive value
TME: Total mesorectal excision
EBRT: External beam radiation therapy
GTV: Gross target volume
TRG: Tumour regression grade
LoG: Laplacian of Gaussian
cT: Clinical tumour
cN: Nodal staging
