# Supplementary material for: Applicability of a pathological complete response magnetic resonance-based radiomics model for locally advanced rectal cancer in intercontinental cohort
Source: Radiat Oncol. 2022 Apr 15;17:78. doi: 10.1186/s13014-022-02048-9 (PMC9013126; doi:10.1186/s13014-022-02048-9)
Supplement: Supplementary file 1 — Additional file 1. Confusion matrices of the predictive performances for the intercontinenental cohort. [file 13014_2022_2048_MOESM1_ESM.docx]

**Supplementary Materials**

*Table 1 - Confusion Matrix for the whole cohort*

|  | Actual 0 | Actual 1 |
| --- | --- | --- |
| Predicted 0 | 32 | 3 |
| Predicted 1 | 18 | 7 |

*Table 1 - Confusion Matrix for the 1.5 T cohort*

|  | Actual 0 | Actual 1 |
| --- | --- | --- |
| Predicted 0 | 9 | 1 |
| Predicted 1 | 12 | 5 |

*Table 2 - Confusion Matrix for the 3T cohort*

|  | Actual 0 | Actual 1 |
| --- | --- | --- |
| Predicted 0 | 23 | 2 |
| Predicted 1 | 6 | 2 |
